# Harvesting nature's bounty: exploring the ethnobotanical landscape of wild edible plants in the Awi Agäw community, Northwestern Ethiopia

**DOI:** 10.1186/s13002-024-00696-7

**Published:** 2024-05-30

**Authors:** Amare Fassil, Ethiopia Mazengia, Bekele Gebreamanuel, Yitayih Dessie, Bulti Kumera, Belsti Atnkut, Destaw Mullualem, Alemu Tsega, Patrick Van Damme

**Affiliations:** 1 Department of Biology, College of Natural and Computational Sciences, Injibara University, Injibara, Ethiopia; 2 Present Address: Faculty of Tropical AgriSciences, Czech University of Life Sciences, Prague, Czech Republic

**Keywords:** Edible plants, Ethnobotany, Indigenous knowledge, Food security

## Abstract

**Background:**

Feeding the world's future population while still facing a variety of socioeconomic and climate change scenarios with uncertain outcomes is a key global societal concern that should be addressed in a science-based manner. Ethiopia boasts a great diversity of wild edible plant species (WEPS), but millions of its citizens still suffer from chronic hunger every year. In this context, we here document the use and conservation of WEPS in the Awi Agäw community, Northwestern Ethiopia.

**Methods:**

We performed a cross-sectional study from October 2021 to June 2022. Ethnobotanical and conservation status data were collected via semi-structured interviews, focus group discussions, field walks and market surveys. A total of 374 respondents from three districts (Guangua, Jawi and Ankasha) were purposely selected for the study. Descriptive statistics, including preference rankings, frequencies and direct matrix rankings, were employed for the data analysis.

**Results:**

We identified a total of 39 WEPS plant taxa distributed among 26 families and 32 genera. The Moraceae and Rosaceae had the greatest numbers of plants, with five and three species, respectively. The WEPS are regularly consumed in the study area to alleviate hunger. However, threats such as habitat loss, agricultural expansion, deforestation for firewood and other reasons, and pesticide use threaten WEPS availability.

**Conclusion:**

Therefore, community-based conservation interventions need to be encouraged to safeguard WEPS and associated traditional knowledge. Furthermore, nutritional quality analysis is recommended for the selection of promising WEPS candidates.

## Introduction

Wild edible plants (WEPs) are defined as ‘species that are neither cultivated nor domesticated or are not actively managed by humans, but are available from their wild natural habitat and used as sources of food’ [1–5]. According to numerous ethnobotanical studies, using WEPs as alternative food sources is a common practice worldwide [1, 3, 4, 6–8]. WEPs are often sourced by local, traditional communities because they are locally available, whereas their use is based on traditional ethnobotanical knowledge that has been accumulated, tested and validated over centuries of use, with low costs involved in sourcing them (usually only collection work). WEPs are considered to provide great benefits to vulnerable, poor populations [4, 9, 10]. WEPs bridge food gaps during times of drought or seasonal food scarcity (e.g., tiding-over periods) [1, 8, 11, 12]. The WEPs can be directly used for snacks and/or side dishes [10, 13] but also presents an opportunity for trade and can thus generate additional income and improve livelihoods if properly supported by extension services [14, 15] and an overall enabling environment. However, the problem is that ethnobotanical and associated indigenous knowledge is rapidly disappearing and should thus be documented and shared for future WEP nutritional value analyses and use recommendations. In combination with the current conservation status data of prospective WEPs, studies may also inform sustainable environmental stewardship [8, 16, 17].

The varied topography and wide spectrum of habitats of Ethiopia blesses the country with diverse natural resources, such as land, soil, forests, water and wildlife, which provide numerous im/material ecosystem services and represent the fundamental resource base for maintaining and improving livelihoods [1, 18]. The country is an important wild and cultivated plant genetic diversity hotspot that can form the basis for developing a more sustainable food provision system [19]. However, the country is also one of the world’s nine countries that will represent half of the projected population growth between 2019 and 2050, bringing additional challenges to efforts to eradicate poverty and hunger [20]. Thus, natural resources such as WEPs could be indispensable factors in addressing poverty and hunger alleviation. However, the consumption of WEPs in Ethiopia is currently a widespread coping mechanism that may come under pressure from increasing population numbers, especially in drought-prone areas [10, 14, 21, 22].

However, even though there is rich WEPs diversity in the country, there is very limited and only fragmentary formal ethnobotanical information on the cultural and socioeconomic values of Ethiopian plants [1, 8]. Still many more wild species are believed to be edible and undocumented yet [23]. For example, the Awi Administrative Zone is among the 11 administrative zones of the Amhara Regional State, where few ethnobotanical studies on WEPs have been performed. To our knowledge, there has been only one previous ethnobotanical study on WEPs in the zone that was performed in Banja and Guangua districts [24]. Despite this, the study area is under pressure of declining forest resources alarmingly and associated indigenous knowledge. There is also a lack of prior ethnobotanical research conducted in Jawi and Ankasha districts, where the current study took place. Hence, documenting ethnobotanical knowledge related with WEPs use and management practices before persistence loss is a well-timed and vital activity.

Given this context, our study was guided by several key scientific questions that will be answered: (1) which plant species do local communities use for food? (2) Are there WEPs community preferences regarding plant growth forms, plant parts used, modes of preparation and times of use? (3) What are the conservation threats to these WEPs? As a result, the objective of current study was (1) to identify and document ethnobotanical knowledge on WEPs consumption and perception for use, (2) identify the economic contribution of WEPs and its implication to livelihood and (3) identify the threats on WEPs conservation and future use strategies. It is expected that policymakers and other stakeholders could use the study's findings to identify and design plans for future use in fighting hunger and poverty.

## Methods

### Study area

**Fig. 1:**
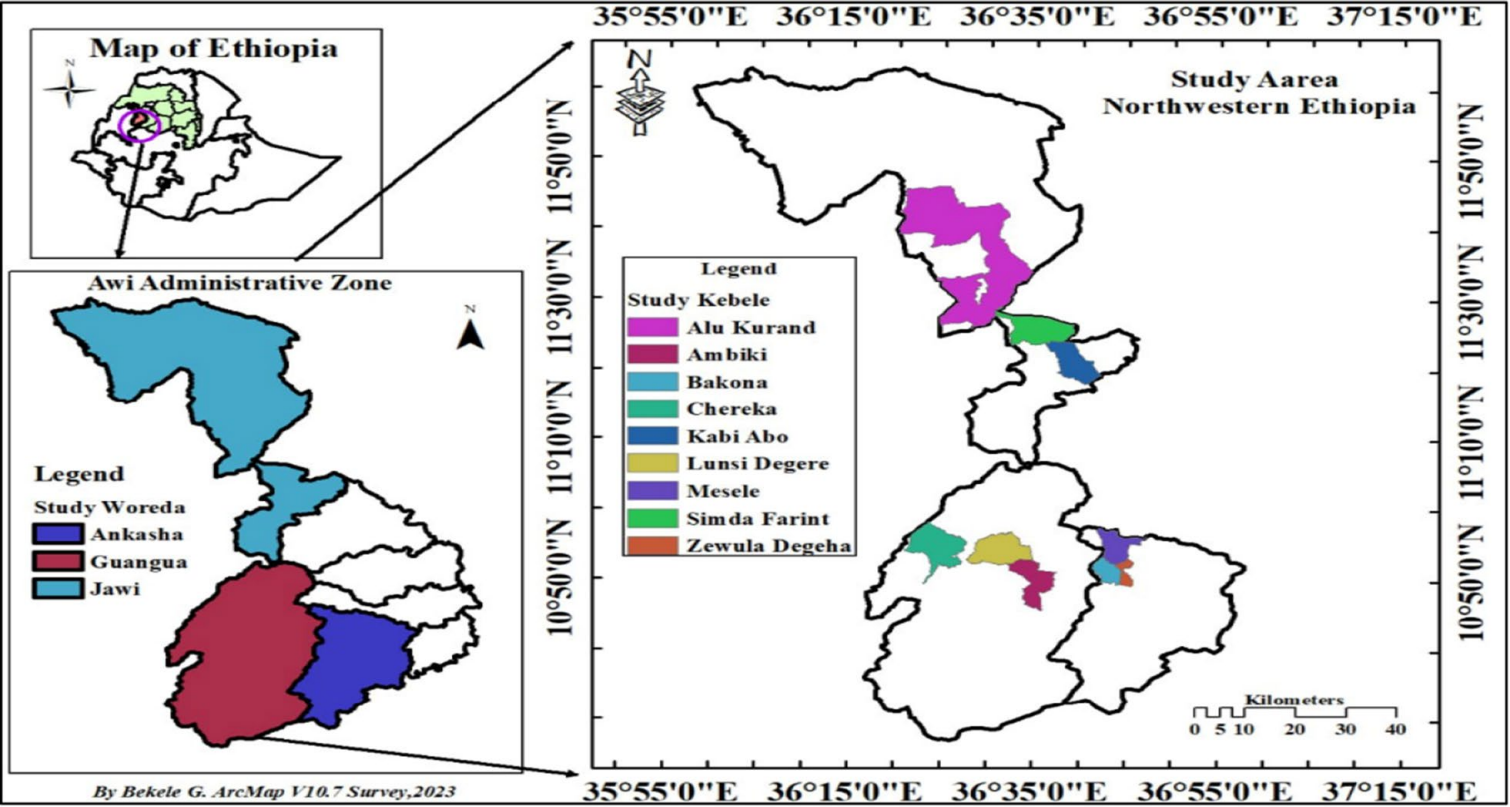
Map of the study area (ArcMap 10.7)

The research was performed in the districts of Jawi, Ankasha and Guangua, which are part of the Awi Administrative Zone, Amhara National Regional State, Ethiopia (Fig. 1). Districts were selected based on their distinctive agro-ecological characteristics, i.e., Jawi district represents moist kola agroecology with an altitude ranging from 500 to 1500 m above sea level (masl) and mean annual precipitation ranging from 1400 to 900 mm, whereas Guangua and Ankasha districts represent Wet Weyna Dega agroecology with altitudes ranging from 1500 to 2300 masl and 1500 to 2800 masl, respectively, and mean annual precipitation > 1400 mm [25]. Geographically, the Jawi district is located between 10° 38′–11° 30′ N and 36°–37° E. The Ankasha district is located between 10° 31′ 46″ and 10° 41′ 32″ N and between 36° 36′ 18″ and 36° 59′ 33″ E, whereas the Guangua district occurs between 10° 57″–10° 95″ N and 36° 30′–36° 50″ E.

### Study communities

Agäws, which migrated due to pressure from the Aksumite Kingdom, ultimately settled in scattered territories and separated into different dialectal groups. The Awi Agäw form one of these groups. The latter still follow the traditions of their ancestors—the seven ‘brothers’ came from Lasta/Seqota to constitute what are now known as the ‘Seven House Agäw’. These seven houses are called after the seven brothers: Banja, Ankasha, Kwakwra, Chara, Metekel, Zigem and Azena [26].

Language, traditional music, indigenous beliefs, horsemen's associations, horse plow traditions and other cultural components have been preserved by the Awi Agäw [26]. The Awi Agäw community communicates in Agäw (Awgni), an Agäw language belonging to the Cushitic subfamily within the Afro-Asiatic linguistic group [27], alongside Amharic, the national language of Ethiopia. People produce house utensils and fly whisks from highland bamboo (*Oldeania alpina*) and horse tails, respectively. These handcrafted items have great sociocultural importance for local communities. Bamboo goods are used to make domestic furniture, musical instruments, basketry and storage bins, traditional sticks and traditional house constructions. The fly whisk (*chira*) handicraft is made by cutting a horse’s tail for ceremonial use by older men and priests during cultural festivities and holidays, whereas it is also used by older horsemen when they ride horses and conduct traditional ceremonies in their respective localities [28].

### Climate

**Fig. 2:**
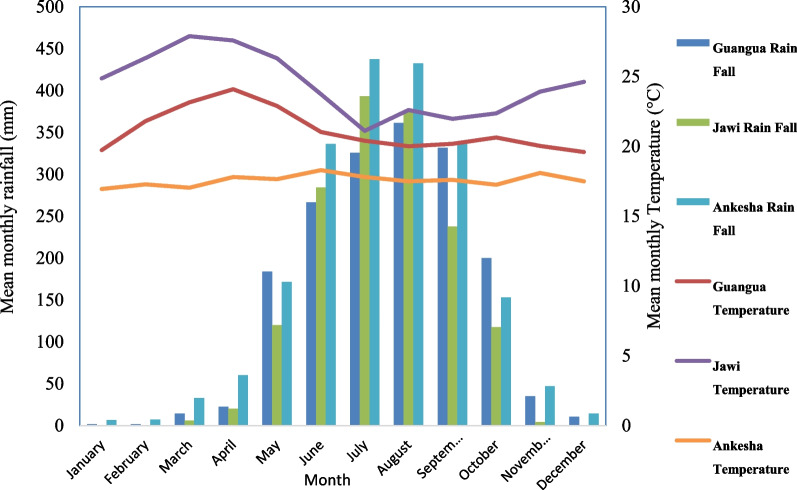
Mean monthly rainfall and temperature of the Jawi, Guangua and Ankasha districts [30]

Based on Ethiopia’s climatic zone typology, the study area falls under moist kola and Wet Weyna Dega agro-ecological zones [29]. In the three research districts, > 90% of the total annual rainfall is recorded from early May to October (Fig. 2), with a peak value occurring in August (i.e., 390 mm). At the district level, the highest annual rainfall is recorded for the Ankasha district, followed by the Guangua and Jawi districts, with values of 2037.77 mm, 1755.78 mm and 1958.19 mm, respectively (Fig. 2).

### Sampling design and informant selection

Ethnobotanical data were collected using the participatory rural appraisal approach in two contrasting seasons, from October to December 2021 and April to June 2022, when most WEPs are available after long and short rainy seasons, respectively. The Guangua, Jawi and Ankasha districts were purposively selected based on their known/documented natural resource potential and agro-ecological representativeness after proper discussions with local agricultural experts. Similarly, study kebeles (a kebele is the lowest administrative unit in Ethiopia) selected purposively based on WEPs resource availability, infrastructure access for ease of data collection and based on recommendations given by Kebele administrators and Agricultural officers. Hence, from Guangua district, we selected Ambiki, Chereka and Lunsi Degere kebeles. Similarly, from the Jawi district, Alu Kurand, Kabi Abo and Simda Farint kebeles, and from the Ankasha district, Mesele, Bakona and Zewula Degeha kebeles were selected. The respondents at the district and kebele levels were selected proportionately to collect both qualitative and quantitative data via semi-structured interviews, key informants, focus group discussions (FGDs) and guided field walks. A total of 36 key informants, i.e., 12 per district, were selected to collect data on WEPs types, parts used and modes of consumption, multipurpose uses of WEPs and their market potential. Key informants were selected based on recommendations given by local elderly persons, kebele administrative bodies and Development Association (DA) officers.

Additionally, three focus group discussions (FGDs), one FGD per district and each with 10 members (for a total of 30 participants), were organized to obtain information on threats to WEPs conservation and the marketability of WEPs. The focus group discussions (FGDs) participants included kebele administrators, religious leaders, agricultural experts and schoolteachers. Focus group discussions (FGDs) members were asked to provide a list of WEPs sold and current market prices in the Jawi, Chagni and Azena marketplaces in the Jawi, Guangua and Ankasha districts, respectively.

The proportional sample size allocation method was used to assign the remaining 308 respondents across the three districts (i.e., Guangua district, *n* = 135; Ankasha district, *n* = 123; and Jawi district = 50) for the semi-structured interviews [31]. Respondents for semi-structured interviews were randomly selected from the study districts by subsequently walking through the 'streets,' starting from the first inhabitant of contact until the required number was attained. The interviewees were asked to provide a full list of plant species used for food, their perceptions of their respective WEPs use, their frequency of use, etic categorization based on factors such as geographic location, ethnic background, dietary habits, or economic status in relation to their knowledge and use of the WEPs, and emic categorization such as nutritional value and seasonal availability, along with their reasons for consumption. Key informants were selected to try and document community preferences for different WEPs utilizations, multipurpose uses of WEPs and their market potential. FGDs and guided field walks were organized to provide information on the threats encountered for/in WEPs conservation and the reasons why the communities are not highly engaged in WEPs consumption and/or commercialization.

### Plant specimen collection and identification

**Fig. 3:**
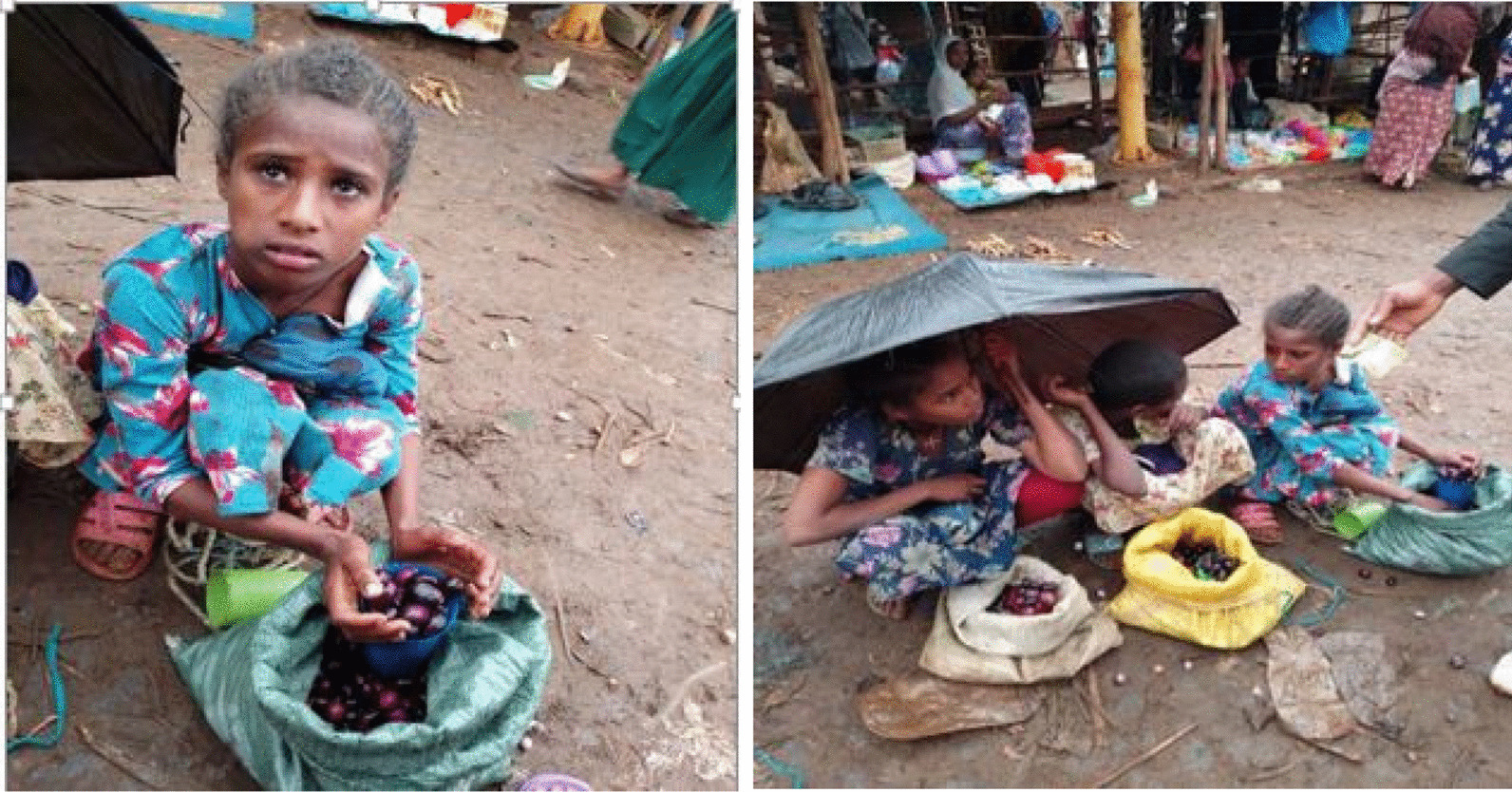
Teenagers selling Syzygium guineense fruits at the Chagni local market (Photo by Amare Fassil, June, 2023)

Plant specimens were collected during field surveys. All field and laboratory protocols were approved by the Injibara University College of Natural and Computational Sciences Institutional Review Committee in accordance with international laws such as the Declaration of Helsinki and the ethical clearance guidelines developed by the Ethiopian Science and Technology Agency. While collecting plant specimens and seeds from both governmental forests and private lands, permission for collection was obtained from the Awi Administrative Zone Agricultural Office and landowners. Furthermore, informed consent was obtained for the personal pictures shown in Fig. 3.

We also recorded the local names of the species to aid in further taxonomical identification using the Flora of Ethiopia and Eritrea, Volumes 2–7 [32–39]. The full scientific names, families and growth forms were recorded following Martin [40]. All 39 plant species specimens were identified by the first author. Voucher specimens were collected, processed and stored in the Injibara University Herbarium.

### Data analysis

Ethnobotanical data collected through interviews, key informants and FGDs were analyzed through descriptive statistics using Excel spreadsheets and R software (R version 4.1.0). The results are presented in tables and figures and summarized into botanical families, growth forms, use categories and parts following Cotton [41] and Martin [40]. Similarly, priority ranking was performed to identify threats to WEPs conservation and future use; direct matrix rankings [40] were used to analyze the degree of importance of the wild plant species for multiple purposes. Jaccard’s coefficient of similarity (JI) was calculated to evaluate WEPs compositions and degrees of similarity from studies done with similar or adjacent floristic regions and agroecology [42].

$$\text{JI}= \frac{c}{a+b-c}$$

where *a* is the total species in the study area; *b* is the total species in the reference area; and *c* is the number of common species between the study area and the reference.

## Results and discussion

### Demographic characteristics of study area

There were 374 participants in this study out of which 308 of them are ordinary respondents; 30 of them are FGD members and 36 of them are key informants (Table 1). These study respondents’ ages ranging from 18 to 71 and highest number of respondents were within 45–69 age range. Most of the respondents (83.77%) were Orthodox Christianity followers followed by Muslims (15.58%). Based on the vegetation types of Ethiopia, both Ankasha and Guangua districts belonged to dry evergreen Afromontane forest while Jawi district belonged to Combretum/Terminalia broadleaved deciduous woodland type [43].

**Table 1:** Demographic characteristics of the study area indicating geographic coordinates of study kebeles, number, age category and religion study participants and vegetation types and number of inhabitants of Guangua, Jawi and Ankasha districts

No	Study districts	No. of inhabitants	Study Kebeles	Geographic coordinates of study Kebeles			Study Participants			Age category				Religion				Vegetation type
				Latitude	Longitude	Altitude	Respondents	FGD	Key Informants	18–30	31–44	45–69	≥ 70	Orthodox	Protestant	Catholic	Islam	
1	Guangua (*n* = 135)	223,066	Ambiki	10° 50′ 28.40″	36° 35′ 54.94″	1500–2300	55	10	12	7	34	14	–	54	1	–	–	dry evergreen Afromontane forest
			Chereka	10° 54′ 49.23″	36° 24′ 53.23″		57			4	9	43	1	10	–	–	47	
			Lunsi Degere	10° 54′ 24.94	36° 32′ 1.58″		23			3	8	9	3	22	1	–	–	
2	Jawi (*n* = 50)	79,090	Alu Kurand	11° 30′ 11.62″	36° 32′ 40.75″	500–1500	18	10	12	4	10	4	0	18	–	–	–	Combretum- Terminalia broadleaved deciduous woodland
			Kabi Abo	11° 20′ 51.05″	36° 38′ 30.40″		17			5	10	2	0	17	–	–	–	
			Simda Farint	11° 25′ 18.30″	36° 35′ 12.65″		15			5	7	2	1	15	–	–	–	
3	Ankasha (*n* = 123)	199,826	Mesele	10° 55′ 4.57″	36° 43′ 41.05″	1500–2800	55	10	12	1	32	22	0	55	–	–	–	dry evergreen Afromontane forest
			Bakona	10° 51′ 31.41	36° 42′ 42.83		46			0	10	36	0	45			1	
			Zewula Degeha	10° 50′ 1.99″	36° 45′ 18.05″		22			2	11	9	0	22				
Total							308	30	36	31	131	141	5	258	2		48	

### Taxonomic diversity

A total of 39 WEPs distributed across 26 families and 32 genera were identified in the present study indicating a significant level of biodiversity within the study area (Table 1). Comparable results were reported elsewhere in the country [24, 44, 45]. This biodiversity is crucial for ecosystem stability and resilience, offering various ecological services such as soil fertility, air quality and habitat for wildlife. However, the number of WEPs identified in this study is somewhat lower than those from studies performed in Bulen district, northwest Ethiopia [46] and the drylands of the country [22], with 77 and 88 WEPs, respectively.

The Moraceae family had the greatest number of species (6), followed by the Rosaceae family (3), whereas the Anacardiaceae, Apocynaceae, Fabaceae, Malvaceae, Solanaceae and Urticaceae families were represented by two species each. The remaining 18 families were represented by a single species (45% of the total number). The number of WEPs demonstrate that the environment offers a diverse set of species [47].

### Growth forms, plant parts used and mode of consumption

Our study revealed four WEPs growth forms, i.e., trees, shrubs, herbs and climbers, following Kent [48]. Trees (46.15%) and shrubs (41.03%) jointly made up the highest proportion (87.18%) of the WEPs documented. Fruits, leaves, roots, young shoots, gum and nectar are plant parts and/or plant products used for food. Fruits were the most widely used part harvested from 36 WEPs. The diverse growth forms (trees, shrubs, herbs, climbers) and parts used (fruits, leaves, roots, etc.) suggest that these plants play multiple roles in the ecosystem, supporting different organisms and ecological functions.

Most of the reported WEPs were also mentioned in other studies in the country. Thus, 12 species overlap with those from studies in Derashe and Kucha districts (southern Ethiopia) and Bulen districts [46, 49], 16 species overlap with those from Chilga district [45], and 15 species overlap with those from Nech Sar National Park [50]. The use of comparable WEPs nationwide might suggest that people share usage practices and commonalities in WEPs due to ecological adaptations to diverse environmental factors [45].

Among the different WEPs, *Carissa spinarum*, *Cordia Africana, Ficus sur*, *Ficus vasta*, *Syzygium guineense*, *Ximenia Americana* and *Rumex nervosus* were reported from more than 15 districts of the country. The widespread utilization of WEPs, as indicated by multiple citations in Table 2, suggests their ecological importance and potential impact on local ecosystems. Contrary to this, *Senegalia mellifera*, *Ficus laurifolia*, *Pittosporum viridiflorum* and *Zizyphus mucronata were* not previously reported in Ethiopia for their WEPs uses (Table 2).

**Table 2:** Wild edible plants used in Guangua, Jawi and Ankasha districts and their previous WEPs use reports

No.	Scientific name	Family name	Local name	Growth types	Plant parts used	Modes of consumption	Citation in Ethiopia	Voucher number
1.	*Abelmoschus esculentus (*L.) Moench	Fabaceae	*Kema* (Amh)	Herb	Fruit	The fruit is eaten raw	[46, 51]	GY003
2.	*Acacia abyssinica* Hochst.ex.Benth	Fabaceae	*Girar* (Amh)	Tree	Gum	The bark is chewed and juice is swallowed	[52–56]	AM002
3.	*Acanthus sennii* Chivo	Acanthaceae	*Kusheshle* (Amh)	Shrub	Nectar	Nectar juice is sipped by lip	[46, 52, 54, 56–59]	AM001
4.	*Acokanthera schimpe*ri (A.DC.) Schweinf	Apocynaceae	*Bitry*	Tree	Fruit	The fruit is eaten raw	[59–63]	GA014
5.	*Capparis tomentosa (*Forssk.) Edgew	Capparidacee	*Gumero* (Amh)	Shrub	Fruit	The fruit is eaten raw	[1, 53, 55, 64, 65]	JA003
6.	*Carissa spinarum* L	Apocynaceae	*Atsiri*	Shrub	Fruit	The fruit is eaten raw	[1, 8, 46, 52–54, 56–64, 66–70]	JK036
7.	*Cordia africana* L	Boraginaceae	*Bugitsi*	Tree	Fruit	The fruit is eaten raw	[1, 22, 23, 43, 46, 52–64, 66, 67, 70, 71]	JA001
8.	*Dioscorea prahensilis* Benth	Dioscoreaceae	*Sinsa*	Climber	Root	Root tuber is boiled, pilled and eaten	[55]	JA002
9.	*Diospyros abyssinica* (Hiern) F.White	Ebenaceae	*Serkuni*	Tree	Fruit	The fruit is eaten raw	[1, 43, 55, 62, 64]	GY002
10.	*Dovyalis abyssinica* (A.Rich.) Warb	Salicaceae	*Koshim*	Shrub	Fruit	The fruit is eaten raw	[1, 8, 22, 52–54, 56–61, 65, 66, 70]	JK039
11.	*Embelia schimperi* Vatke	Primulaceae	*Enkoku*	Shrub	Fruit	The fruit is eaten raw	[1, 52, 54, 58, 60, 61, 64, 66]	GA009
12.	*Ficus laurifolia* Lam	Moraceae	*Wume*	Tree	Fruit	The fruit is eaten raw	Not Reported	GA006
13.	*Ficus palmata* Forssk	Moraceae	*Beles* (Amh)	Tree	Fruit	The fruit is eaten raw	[1, 8, 56, 62, 66, 70]	GA005
14.	*Ficus sur* Forssk	Moraceae	*Bizari*	Tree	Fruit	The fruit is eaten raw	[1, 8, 22, 46, 52–56, 58–60, 64, 66, 67, 69, 70]	GA004
15.	*Ficus sycomorus* L	Moraceae	*Bannbi*	Tree	Fruit	The fruit is eaten raw	[1, 8, 22, 31, 52, 54–56, 59, 60, 64–68, 72]	GA003
16.	*Ficus vallis-choudae* Delile	Moraceae	*Boba*	Tree	Fruit	The fruit is eaten raw	[1, 67]	GA007
17.	*Ficus vasta* Forssk	Moraceae	*Banbur*	Tree	Fruit	The fruit is eaten raw	[1, 22, 31, 52–54, 56, 58–60, 62–69]	GA008
18.	*Gardenia ternifolia* Schumach. & Thonn	Rubiaceae	*Gambilo*	Shrub	Fruit	eating the fruits directly	[1, 22, 46, 51, 55, 60, 62–64]	GL001
19.	*Grewia villosa* Willd		*Tsannkewi*	Shrub	Fruit	The fruit is eaten raw	[1, 63, 66–69, 71]	GA002
20.	*Lannea schimperi* (Hochst. ex A.Rich.) Engl	Anacardiaceae	*Dugini*	Tree	Fruit	The fruit is eaten raw	[1, 63, 69, 70]	GA001
21.	*Lantana camara* L	Verbenaceae	*Afkoli*	Shrub	Fruit	The fruit is eaten raw	[1, 8, 14, 62, 64, 65]	JK041
22.	*Mimusops kummel* Bruce ex A.DC	Sapotaceae	*Enkui*	Tree	Fruit	The fruit is eaten raw	[1, 8, 22, 46, 52, 56, 58–64, 66, 67, 73]	JK040
23.	*Oxytenanthera abyssinica* (A.Rich.) Munro	Poaceae	*Enkui*	Tree	Fruit	The fruit is eaten raw	[1, 46, 51, 64]	JK037
24.	*Phoenix reclinata* Jacq	Arecaceae	*Selien* (Amh)	Shrub	Fruit	The fruit is eaten raw	[1, 22, 46, 52, 54–57, 59, 60, 64, 67]	JA015
25.	*Physalis peruviana* L	Solanaceae	*Awut*	Herb	Fruit	The fruit is eaten raw	[1, 8, 46, 52, 56, 57, 59, 60, 63, 66, 70]	AM005
26.	*Pittosporum viridiflorum* Sims	Pittosporaceae	*Dengay seber*	Tree	Fruit	The fruit is eaten raw	Not Reported	AM004
27.	*Rhus vulgaris* Meikle	Anacardiaceae	*Ashkambo*	Shrub	Fruit	The fruit is eaten raw	[1, 46, 52, 54, 56, 59, 63, 65, 70]	GY001
28.	*Rosa abyssinica* R.Br. ex Lindl	Rosaceae	*Gimtsi*	Shrub	Fruit	The fruit (hip) is eaten after separated from seeds	[1, 8, 52–54, 56–58, 60, 61, 66]	GA010
29.	*Rubus apetalus* Poir	Rosaceae	*Enjori*	Shrub	Fruit	The fruit is eaten raw	[1, 54, 57–60, 62, 64, 67]	GA011
30.	*Rubus volkensii* Engl	Rosaceae	*Enjori*	Shrub	Fruit	The fruit is eaten raw	[1, 66]	GA012
31.	*Rumex nervosus Vahl*	Polygonaceae	*Embuancho (*Amh.)	Shrub	Shoot	Young shoots pilled and eaten raw	[1, 8, 14, 22, 52–54, 56–60, 62, 64, 66]	JA004
32.	*Senegalia mellifera* (Benth.) Seigler & Ebinger	Fabaceae	*Hanguri*	Shrub	Fruit	The fruit is eaten raw	Not reported	AM003
33.	*Solanum nigrum*	Solanaceae	*Awut* (Amh)	Shrub	Fruit	The fruit is eaten raw	[1, 8, 23, 46, 52, 53, 56, 58, 59, 65, 70, 72, 74]	GY005
34.	*Syzygium guineense* (Willd.) DC	Myrtaceae	*Bahusti*	Tree	Fruit	The fruit is eaten raw	[1, 8, 22, 23, 43, 46, 52, 54, 55, 58–64, 66, 67, 70, 75]	JK038
35.	*Tribulus terrestris* L	Zygophyllaceae	*Kurinchit*	Herb	Fruit	The fruit is eaten raw	[1, 53, 76]	GY006
36.	*Urtica simensis Hochst. ex A.Rich*	Urticaceae	*Sama* (Amh)	Herb	Leaves	Leaves are eaten after cooking with Ethiopian traditional food called *‘Kita’*	[1, 52, 54, 56–60, 62, 66]	AM006
37.	*Vepris dainellii* (Pic.Serm.) Kokwaro	Urticaceae	*Gulmasty*	Tree	Fruit	The fruit is eaten raw	[54]	GA013
38.	*Ximenia americana* L	Olacaceae	*Enkoy*	Tree	Fruit	The fruit is eaten raw	[1, 8, 14, 23, 43, 51, 56–58, 62, 64–70, 72]	GL004
39.	*Zizyphus mucronata* Wild	Rhamnaceae	*Foch*	Tree	Fruit	The fruit is eaten raw	Not Reported	GY004

### Jaccard’s coefficient of similarity index (JI)

Jaccard’s coefficient of similarity index (JI) used to show the degree of similarity of WEPs between the current study and previous studies done in Gojam and Gondar floristic regions (Table 3). Jaccard’s coefficient of similarity index (JI) showed that the study area has the highest (33.33%) species overlaps with Sedie Muja district [52] followed by Tach Gayint district with 31.58% species overlaps [56]. Both Sedie Muja and Tach Gayint districts have three climatic zones, namely, Dega (above 2500 m), Woinadega (2500–1800 m) and Kola (below 1800) which are also similar with study area [52, 56]. The higher species overlaps of the study area with Sedie Muja and Tach Gayint districts may be attributed with similarities in agroecology with the study area. However, slight (5.06%) species overlaps observed between the study area and Metema district which may be attributed with agro-ecologic dissimilarity [43].

**Table 3:** Jaccard’s coefficient of similarity index of the study area on degree species similarity with other study areas in Amhara region

No.	Study areas	Total species number (*a*/*b*)	Common species (*c*)	Jaccard’s coefficient Index (JI)	Percentage of similarity (%)	References
1.	Guangua, Jawi, and Ankasha	39	–	–	–	Study area
2.	Yilmana Densa and Quarit	32	15	0.2679	26.79	[58]
3.	Sedie Muja	33	18	0.3333	33.33	[52]
4.	Quara	36	10	0.1538	15.38	[55]
5.	Tach Gayint	36	18	0.3158	31.58	[56]
6.	Ensaro	43	17	0.2615	26.15	[66]
7.	Metema	44	4	0.0506	5. 06	[43]

### Perception of local communities on WEPs use

A total of 374 respondents participated in the assessment of current local community WEPs use. Two hundred five of them (54.8%) responded that they received income from them to support their family in addition to household consumption. The rest of the participants, on the other hand, either did not earn enough money to sustain their families (*n* = 66; 17.6%) or did so only with extreme difficulty (*n* = 103; 27.5%). Respondents were asked whether there had been incidents of food shortage in their locality during their life. Approximately half (*n* = 187; 50%) answered positively. On the other hand, 159 (42.5%) respondents said they had not experienced any food shortage in their community, whereas 28 (7.5%) had no clear idea.

WEPs collection and consumption are currently vital practices in local communities. However, there was a response variance among respondents on WEPs consumption rates when food scarcity was mentioned. Of the 374 respondents, 218 (58.29%) said they used WEPs during food shortages, while the remaining 156 (41.71%) did not think that WEPs might be considered a solution to cover food shortages. However, 89% (*N* = 335) of respondents reported consuming WEPs for reasons other than food scarcity, such as its use as a supplement and for therapies. Furthermore, our research revealed that the majority of respondents (88.5%) had either had prior WEPs use experience or had at least witnessed them. In terms of WEPs consumption frequency, 170 (45.45%) respondents confirmed that they consumed WEPs extremely frequently, whereas 196 (52.41%) respondents ate WEPs on a regular basis. Only 2.13% of responders only occasionally harvested WEPs. Our study demonstrated that WEPs intake in the study area is a daily activity that is widely acknowledged to be highly relevant.

The respondents were requested to answer the question ‘In which circumstances does the community use WEPs?’ Our study revealed that community members consume WEPs during the drier periods of the year (especially children, *N* = 186, 49.73%), during prolonged drought and famine (*N* = 100, 26.74%) and as emergency food in food-insecure conditions (*N* = 57, 15.24%) and for other unspecified reasons (*N* = 31, 8.29). WEPs consumption for supplementary, emergency and seasonal conditions was confirmed by a comprehensive study performed by Lulekal, Asfaw [1] in the country.

### Economic contribution and marketability of WEPs

Local communities fulfill their socioeconomic needs by selling crop products such as sorghum, maize and teff, as well as animal products such as cattle, horses, sheep and goats. Additionally, they engage in charcoal production, local trade, including traditional crafts and market transactions, and the sale of wild edible plant (WEPs) products, either individually or in combination with two or more of the aforementioned activities. Selected WEPs were observed to be sold at local markets and around school gates (Fig. 3).

*Syzygium guineense*, *Oxytenanthera abyssinica* and *Dioscorea prahensilis* were the top three WEPs frequently mentioned for sale in local markets (Table 4). Similarly*, D. prahensilis* had the highest mean market price of 30.20 Ethiopian Birr (ETB)/kg. *D. prahensilis* produces root tubers from May to early June, when there is limited rain in the region (Jawi district) and little abundance, which may cause the price to be relatively higher than that of the other marketed WEPs. The sale of WEPs provides a source of income for many families, relying on this income in addition to household consumption. This economic aspect is crucial in regions where other reliable income sources might be limited. However, the widespread utilization and economic reliance on WEPs sale, may put pressure on these species, leading to overharvesting. As a result, special conservation policy should be employed for sustainable use of aforementioned WEPs.

**Table 4:** Mean market price and frequency of citation on WEPs sale for livelihood

No.	Plant species	Number of citations (*n* = 45)	Citation percentage (%)	Mean Market price/kg (ETB)
1.	*Cordia africana*	3	2.54	5.44
2.	*Dioscorea prahensilis*	24	20.34	30.20
3.	*Ficus laurifolia*	4	3.39	6.00
4.	*Ficus sur*	6	5.09	5.56
5.	*Lannea schimperi*	3	2.54	7.87
6.	*Oxytenanthera abyssinica*	24	20.34	7.61
7.	*Rosa abyssinica*	4	3.39	6.62
8.	*Rubus apetalus*	5	4.24	6.09
9.	*Syzygium guineense*	32	27.12	5.51
10.	*Vepris daniellii*	10	8.47	9.16

### Threats on WEPs conservation and future use

The extent to which human activities pose a threat to WEPs in their natural habitats varies by location and level of impact [46, 49]. The types of threats and their levels of impact on WEPs conservation were identified following Bekele, Woldeyes [77] using the priority ranking method [40]. Ten key informants were selected and asked to list and rank WEPs conservation threats on a scale of 1 to 5 (with 1 being the least destructive and 5 the most destructive threat; following Balemie and Kebebew [49] and Berihun and Molla [46]. A total of five threats to WEPs were identified in the study area (Table 3). In descending order, agricultural expansion and land-use change, deforestation for construction and firewood, and overharvesting/selective harvesting of multipurpose trees were the three most-cited destructive factors (Table 5). Similar studies from elsewhere in the country evidenced comparable threats to WEPs [22, 49, 70]. The threats identified, such as agricultural expansion, deforestation and selective harvesting, further endanger these species and their ecosystems, potentially leading to reduced biodiversity and degradation of natural resources.

**Table 5:** Priority ranking of threats to WEP conservation (Key: *R* = Respondents)

Major threats	R1	R2	R3	R4	R5	R6	R7	R8	R9	R10	Total	Rank
Habitat loss due to roads, forest fires and invasive species	1	2	1	1	2	3	1	2	3	1	**17**	**5**
Agricultural expansion and land-use change	5	4	5	5	4	4	5	4	5	5	**46**	**1**
Deforestation for construction and firewood	4	5	3	4	5	5	4	5	4	4	**43**	**2**
Overharvesting/selective harvesting of multipurpose trees	3	5	4	3	1	2	3	3	2	3	**29**	**3**
Unsustainable usage of insecticides and pesticides	2	3	2	2	3	1	2	1	1	2	**19**	**4**

### Direct matrix ranking for the multipurpose use of WEPs

The 36 key informants (KI) were requested to free list multipurpose uses of WEPs and number of citation by KI was summed up and used as a prioritizing criterion. Top six more frequently mentioned WEPs selected downwards and randomized for fairness. Similarly, five use categories were also filtered out from free listings. Direct matrix ranking was employed to rank these six WEPs (*Cordia africana*, *Zizyphus mucronata*, *Ficus sycomorus*, *Ximenia americana*, *Dioscorea prahensilis*, and *Embelia schimperi*) across the five use categories following Martin [40]. Ranking was carried out based on the opinions of 10 key informants (from the three districts) to assess the relative importance of the six WEPs in the study area over different use categories (Table 6). Five use categories, namely, traditional medicine, wood for fuel, construction materials, fencing and farm utensils, were used to compare the utilization of the selected WEPs, rated from 0 to 4, where 0 signifies not used, 1 indicates least used, 2 reflects good use, 3 implies very good use, and 4 denotes excellent use.

**Table 6:** Direct matrix ranking of six WEPs in five use categories

Plant species	Use categories						
	Traditional medicine	Wood fuel	Construction material	Fencing	Farm utensils	Total	Rank
*Cordia Africana*	0	4	4	2	3	**13**	**3**
*Zizyphus mucronata*	2	4	2	4	3	**15**	**1**
*Ficus sycomorus*	2	4	2	3	1	**12**	**4**
*Ximenia Americana*	1	4	2	3	1	**11**	**5**
*Dioscorea prahensilis*	2	0	3	4	0	**9**	**6**
*Embelia schimperi*	4	4	2	3	2	**15**	**1**
**Total**	**11**	**20**	**15**	**19**	**10**		
**Rank**	**4**	**1**	**3**	**2**	**5**		

The top three ranked use categories reported by respondents for the six WEPs are wood fuel, fencing and construction material, in decreasing order (Table 6). Comparable results for various use categories, such as medicine, construction materials, fuel wood and fencing, were reported elsewhere in the country [45, 46]. Among the six WEPs, *Embelia schimperi* and *Zizyphus mucronata* were the most commonly used plants for multiple purposes followed by *Cordia africana* and *Ficus sycomorus*, respectively. Cosequently, plants such as *Zizyphus mucronata* and *Embelia schimperi*, which ranked highest in versatility, should be prioritized for conservation efforts because of their diverse uses and potential overexploitation.

## Conclusions and recommendation

The study identified 39 wild edible plants (WEPs) across 26 families and 32 genera, reflecting significant diversity, although other studies in the country reported varying numbers of these plants. Trees and shrubs were predominant among the four growth forms identified, with fruits being the most commonly harvested plant part, highlighting the versatility of WEPs in local diets. WEPs collection serves as a vital income source for many local communities, although opinions on its role in food shortages vary. However, most respondents acknowledged using WEPs beyond mere sustenance, including for supplementation and therapeutic purposes.

Local economies benefit from the sale of WEPs products, such as *Syzygium guineense* and *Dioscorea prahensilis*, underscoring their economic importance to local communities indicating their market potential. However, threats such as agricultural expansion, deforestation and overharvesting endanger WEPs conservation, necessitating concerted efforts for sustainable management. Community-based conservation initiatives, alongside awareness programs, can mitigate risks. Promoting high-value WEPs species cultivation and marketing offers additional income opportunities. Collaboration and knowledge sharing among stakeholders can enhance conservation strategies, recognizing the cultural, ecological and economic significance of WEPs. Safeguarding these resources ensures resilience and livelihood security for future generations in the study area. Moreover, policy interventions aimed at protecting biodiversity, conserving natural habitats and promoting sustainable agricultural practices are crucial. Integrating WEPs conservation into broader biodiversity conservation strategies and land-use planning frameworks can help mitigate threats and ensure the long-term viability of WEPs populations.

## Data Availability

The data that support the findings of this research are available from the corresponding author upon motivated request.

## Abbreviations

WEPs: Wild edible plant species
NMA: National Meteorological Agency
FGDs: Focus Group Discussions
DA: Development Association
ETB: Ethiopian Birr
